# Unilateral mechanical asymmetry: positional effects on lung volumes and transpulmonary pressure

**DOI:** 10.1186/2197-425X-2-4

**Published:** 2014-02-05

**Authors:** Gustavo A Cortes-Puentes, Kenneth Gard, Joseph C Keenan, Alexander Adams, David Dries, John J Marini

**Affiliations:** Department of Pulmonary and Critical Care Medicine, Regions Hospital, 640 Jackson St., Saint Paul, MN 55101 USA; Department of Pulmonary, Allergy, Critical Care and Sleep Medicine, University of Minnesota, Minneapolis, MN 55455 USA; Department of Surgery, University of Minnesota, Minneapolis, MN 55101 USA

**Keywords:** Mechanical ventilation, Pleural effusion, Positive end-expiratory pressure (PEEP), Animal model, Positioning, Transpulmonary pressure, Asymmetry, Lung volumes

## Abstract

**Background:**

Ventilated patients with asymmetry of lung or chest wall mechanics may be vulnerable to differing lung stresses or strains dependent on body position. Our purpose was to examine transpulmonary pressure (*P*_TP_) and end-expiratory lung volume (functional residual capacity (FRC)) during body positioning changes in an animal model under the influence of positive end-expiratory pressure (PEEP) or experimental pleural effusion (PLEF).

**Methods:**

Fourteen deeply anesthetized swine were studied including tracheostomy, thoracostomy, and esophageal catheter placement. Animals were ventilated at *V*_T_ = 10 ml/kg, frequency of 15, *I*/*E* = 1:2, and FIO_2_ = 0.5. The animals were randomized to supine, prone, right lateral, left lateral, and semi-Fowler positions with a PEEP of 1 cm H_2_O (PEEP1) or a PEEP of 10 cm H_2_O (PEEP10) applied. Experimental PLEF was generated by 10 ml/kg saline instilled into either pleural space. *P*_TP_ and FRC were determined in each condition.

**Results:**

No significant differences in FRC were found among the four horizontal positions. Compared to horizontal positioning, semi-Fowler's increased FRC (*p* < 0.001) by 56% at PEEP1 and 54% at PEEP10 without PLEF and by 131% at PEEP1 and 98% at PEEP10 with PLEF. Inspiratory or expiratory *P*_TP_ showed insignificant differences across positions at both levels of PEEP. Consistently negative end-expiratory *P*_TP_ at PEEP1 increased to positive values with PEEP10.

**Conclusions:**

FRC did not differ among horizontal positions; however, semi-Fowler's positioning significantly raised FRC. *P*_TP_ proved insensitive to mechanical asymmetry. While end-expiratory *P*_TP_ was negative at PEEP1, applying PEEP10 caused a transition to positive *P*_TP_, suggestive of reopening of initially compressed lung units.

**Electronic supplementary material:**

The online version of this article (doi:10.1186/2197-425X-2-4) contains supplementary material, which is available to authorized users.

## Background

Measurements derived from the airway circuit of the ventilator have served clinicians well in making therapeutic decisions when treating diffuse lung injury. Quite recently, however, transpulmonary pressure (*P*_TP_) - estimated as the difference between airway pressure (*P*_AW_) and esophageal pressure (*P*_ES_) - has been advocated as more physiologically relevant for evaluating the mechanical properties of the lung and chest wall [1–5]. In theory, *P*_TP_ could be a valuable clinical guide to adjust the mechanical ventilator, to evaluate lung compliance, and to monitor the lung's response to specific therapeutic decisions, free of the confounding influence of chest wall and breathing effort. The value of *P*_TP_, however, is predicated on lung uniformity and on its retention of accuracy during position changes. Surprisingly, little information is available regarding the effects of asymmetry and positioning on global or regional pulmonary mechanics.

Absolute values and dynamic tidal changes of *P*_ES_ have been examined in the setting of healthy or symmetrically diseased lungs [1, 2]. Those evaluations have shown that the esophageal balloon catheter reliably measures the pressures that surround its immediate environment. However, the relationship between estimated *P*_TP_ and aerated lung volume is still under discussion. Aerated lung volume, as assessed by functional residual capacity (FRC), as well as volume distribution can be altered by body positioning [6–8]. For example, improved oxygenation during prone positioning has been attributed to improved ventilation/perfusion matching with the possible contribution of increasing FRC [9, 10]. Our purpose was to examine the reliability of *P*_TP_ for characterizing total lung volume changes due to body positioning when the mechanical properties of the thorax are symmetrically and nonsymmetrically distributed.

## Methods

The Animal Care and Use Committee of Regions Hospital (St. Paul, MN, USA) approved this protocol. Fourteen healthy Yorkshire pigs (mean weight 36.6 ± 6 kg) were premedicated with intramuscular Telazol® (tiletamine/zolazepam)/xylazine (2.2 and 6.6 mg/kg, respectively, Zoetis, Inc., Florham Park, NJ, USA), and after tracheostomy, they received inhalational anesthesia with a continuous flow of 0.5% to 2% isoflurane and a 50% oxygen/50% nitrous oxide mixture. The preparation also included femoral venous and arterial catheters and suprapubic cystostomy. A chest tube was inserted with a cephalad orientation into the pleural cavity and all air evacuated. In seven animals, thoracostomies were performed on the right side, and in the remainder, thoracostomy was performed on the left side. When required by the protocol, 10 ml/kg of body temperature saline was instilled to simulate pleural effusion. The tip of an esophageal balloon catheter was advanced to a depth of approximately 40 to 50 cm from the incisors, and the balloon was inflated with 1.5 ml of air. Gastric positioning was confirmed by transient increase in pressure during compression of the abdomen and by gastric content return. The esophageal balloon catheter was then withdrawn to a depth of approximately 30 to 40 cm where obvious cardiac oscillations were observed in the tracing. Inhalational anesthesia was slowly discontinued over approximately 30 min and replaced by a titrated intravenous infusion of Telazol®, ketamine, and xylazine. Adequate depth of anesthesia was ensured by continuously monitoring the bi-spectral index (BIS; Covidien, Carlsbad, CA, USA). Pigs were then ventilated using the Engström Carestation™ (GE Healthcare, Madison, WI, USA): *V*_T_ = 10 ml/kg, frequency titrated to a P_ET_CO_2_ of 30 to 40 mmHg, *I*/*E* of 1:2, positive end-expiratory pressure (PEEP) of 1 or 10 cm H_2_O, and FIO_2_ of 0.5. At the end of the experiment, animals were euthanized by rapid injection of Euthasol® (Virbac Corporation, Fort Worth, TX, USA).

### Experimental protocol

Respiratory system mechanics and FRC were evaluated in response to unilateral pleural effusion (PLEF), PEEP, and positioning. Volume-controlled ventilation (VCV) settings remained unmodified during the experimental protocol except during recruitment maneuvers, which were performed using ten breaths of pressure-controlled ventilation (PCV) with an inspiratory pressure of 40 cm H_2_O and PEEP = 20 cm H_2_O [11]. FRC was measured using a ventilator-integrated modified nitrogen wash-in/wash-out method (Engström Carestation™, GE Healthcare, Madison, WI, USA) [5]. A PEEP of 1 cm H_2_O (PEEP1) is a technical requirement when FRC is measured using the proprietary modified wash-in/wash-out technique (GE Healthcare, Madison, WI, USA) and served as the least value for end-expiratory airway pressure [12]. A PEEP of 10 cm H_2_O (PEEP10) was selected due to its common use in clinical mechanical ventilation settings and its generous distending effect on normal pig lungs (as observed in our previous experience [4, 5]). These PEEP levels were randomly applied during each of the experimental conditions.

Five positions, applied in random order, were examined: semi-Fowler's (inclined 30° from horizontal in the sagittal plane), prone, supine, right lateral, and left lateral. Positional effects on respiratory system mechanics and FRC were evaluated in four experimental conditions: non-PLEF-PEEP1, non-PLEF-PEEP10, PLEF-PEEP1, and PLEF-PEEP10.

After a 10-min stabilization period in a given tested position, *P*_ES_ was recorded at the static end-expiratory and end-inspiratory points of the tidal cycle. After every position variation, baseline conditions (supine position and PEEP1) were reestablished and the esophageal pressure balloon catheter was recalibrated to assure the fidelity of its pressure tracing. Vital signs were monitored during the entire experiment, and all hemodynamic data were recorded 1 min after each alteration of experimental condition. In one representative animal, helical computerized tomography (CT) images of the chest were obtained for each position with unilateral PLEF, using a 64-slice CT scanner (LightSpeed™ VCT, GE Healthcare, Milwaukee, WI, USA).

### Statistical analysis

The effects of positioning, PEEP, and PLEF on the dependent variables of FRC and *P*_TP_ were analyzed by ANOVA, with internal comparisons and contrasts made using the Tukey method (Minitab 2013, Minitab, Inc., State College, PA, USA).

## Results

**Figure 1:**
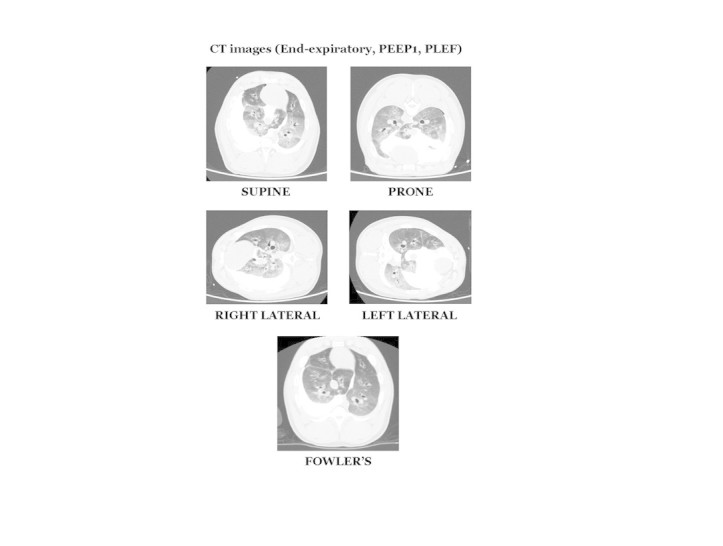
**Computerized tomography imaging of a representative animal for the five evaluated positions.** Note that aerated lung tends to be in the nondependent (uppermost) lung regions. Collapsed or poorly aerated lung is concentrated in the lower, gravity-dependent regions.

A set of computerized tomography images from a representative animal is shown in Figure 1. Five positions are presented at end-expiration with PLEF instilled into the right hemithorax (10 ml/kg of body temperature saline was instilled to simulate pleural effusion). For each studied position, a reduced or absent imaging pattern of aeration in the dependent regions was observed when compared to the nondependent regions of the lung.

### FRC response to position and PEEP

**Figure 2:**
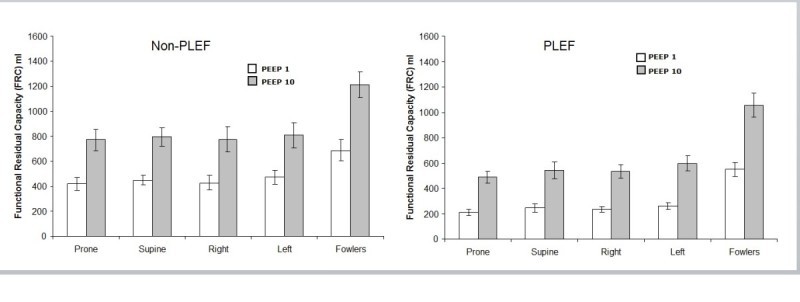
**Mean functional residual capacities (±s.e.m.).** Semi-Fowler's, left lateral, prone, right lateral, and supine positions for PEEP1 and PEEP10 with no pleural effusion (left panel) and with unilateral pleural effusion (right panel). No differences were observed between the four horizontal positions (left, prone, right, supine) with or without pleural effusion at PEEP1 and PEEP10. Semi-Fowler's position significantly increased FRC compared to all horizontal positions with and without pleural effusion at both PEEP levels as identified by asterisks.

Determinations of FRC for each position in all tested conditions are displayed in Figure 2. No significant differences in FRC were seen among the supine, prone, right lateral, and left lateral variations of horizontal position (*p* = NS). For non-PLEF-PEEP1, FRC was significantly increased with semi-Fowler positioning compared to each of the horizontal positions (*p* < 0.001). With PEEP1 applied, mean FRC in semi-Fowler's was 246 ml or 56% greater than mean horizontal FRC. At PEEP10, semi-Fowler's mean FRC was 424 ml or 54% greater than mean horizontal FRC. Among all positions without PLEF, PEEP10 caused a mean FRC increase of 389 ± 170 ml or 79% compared to PEEP1 FRC.

### FRC response to position and PEEP in the setting of pleural effusion

At PEEP1, pleural effusion reduced FRC by 191 ± 136 ml or 39% of non-PLEF FRC (*p* < 0.0001). This volume loss represented 56% of the instilled PLEF volume. Regarding the laterality of PLEF, there was no significant difference in FRC between right PLEF and left PLEF. For PLEF-PEEP1 and PLEF-PEEP10, no differences in FRC were observed among the horizontal positions. Compared to horizontal positions, semi-Fowler's position increased FRC by 131% and 98% for PEEP1 and PEEP10, respectively. PEEP increased mean FRC by 339 ml or 113% with PLEF present.

### Transpulmonary pressure

**Figure 3:**
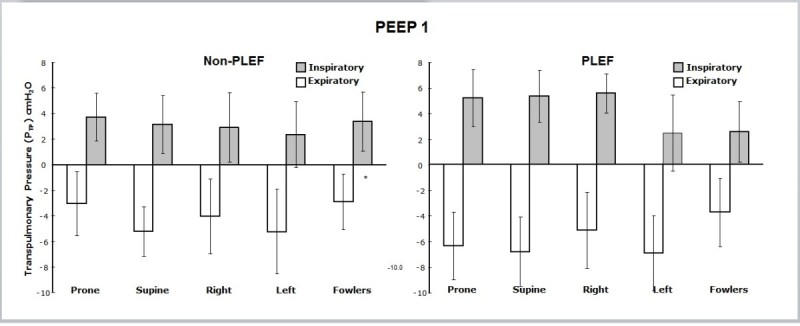
**Mean transpulmonary pressures (±s.d.) for PEEP1.** Semi-Fowler's, left lateral, right lateral, prone, and supine positions (left panel) with and without pleural effusion (PLEF, right panel). End-inspiratory and end-expiratory pressures are displayed. No differences were observed between positions during end-inspiration and end-expiration with or without PLEF. Mean transpulmonary excursion pressure was decreased (*p* < 0.05) in the semi-Fowler's position (asterisk).

Mean *P*_TP_ values for each position in non-PLEF and PLEF at PEEP1 are displayed in Figure 3. End-expiratory *P*_TP_ was consistently negative and similar for each of the five tested positions. End-inspiratory *P*_TP_ at PEEP1 was consistently positive and did not differ statistically among positions. PLEF decreased end-expiratory *P*_TP_ by 1.7 cm H_2_O to -5.8 ± 3.0 (*p* < 0.002), while end-inspiratory *P*_TP_ was statistically unchanged.

**Figure 4:**
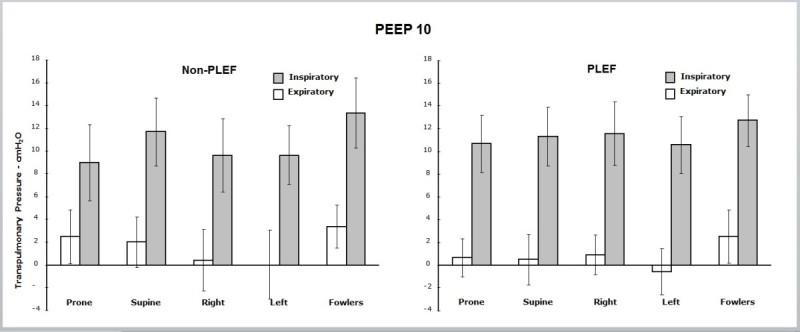
**Mean transpulmonary pressures (±s.d.) for PEEP10.** Semi-Fowler's, left lateral, right lateral, prone, and supine positions (left panel) with and without pleural effusion (PLEF, right panel). No differences were seen between positions with or without PLEF. End-expiratory *P*_TP_ was positive or near zero with PEEP10. PLEF did not significantly change end-expiratory or end-inspiratory *P*_TP_. *P*_TP_ excursion pressures did not differ between position with or without PLEF.

Figure 4 displays *P*_TP_ for the five positions for the non-PLEF and PLEF conditions at PEEP10. Differences with position change were not seen in either condition. Applying PEEP10 consistently increased end-inspiratory and end-expiratory *P*_TP_ for all positions.

In transitioning from PEEP1 to PEEP10 (with or without PLEF), end-expiratory *P*_TP_ increased from a negative pressure to a positive or near-zero pressure for all five positions. *P*_TP_ excursion pressures (the difference between end-inspiratory and end-expiratory *P*_TP_) with PLEF-PEEP1 were similar between positions except for the semi-Fowler's, which had a reduced *P*_TP_ excursion compared to the *P*_TP_ of the other four positions (*p* < 0.05).

## Discussion

The salient findings of this study can be summarized as follows: (1) FRC was higher in semi-Fowler's position but remained relatively unchanged among all horizontal positions, regardless of orientation and despite changes of regional aeration caused by asymmetrical extrapulmonary forces. (2) Applying PEEP10 consistently increased FRC and attenuated the negative values of end-expiratory *P*_TP_ observed at PEEP1 in all positions, regardless of PLEF and its lung volume-compressing effects. (3) Both FRC and *P*_TP_ were insensitive to variations of regional mechanics caused by horizontal position changes, even in this highly nonsymmetrical mechanical setting of unilateral PLEF.

### Semi-Fowler's position vs. FRC

Lung volume is normally affected by position change, especially when subjects assume recumbency from the upright position [6]. Additionally, the global lung environment (pressure gradients, airway closure patterns, perfusion, and ventilation) may be influenced by positional variation [13]. In our study, all the horizontal positions tested showed similar values of global FRC, despite visually impressive redistribution of the aerated gas (Figure 1). Conversely, semi-Fowler's position countered the lung volume reduction associated with recumbency in all tested conditions, presumably by shifting the abdominal content caudally, relieving pressure against the diaphragm.

### PEEP10 and PLEF interactions

As expected from a previous report [5], PEEP10 restored FRC to its non-PLEF baseline in all positions. PLEF generates a pleural hydrostatic pressure and reduces the FRC in all positions; however, our radiographic evidence and previous work support the idea that the lung progressively is lifted above the level of pleural fluid during tidal inflation, and asymmetrical chest expansion redistributes PLEF as airway pressure rises [5]. Applying PEEP10 may attenuate this exaggerated tidal inflation-deflation and/or recruitment and de-recruitment cycle by increasing the baseline airway pressure and lung volume.

### Positioning and transpulmonary pressure

As opposed to spontaneous breathing, positive-pressure ventilation increases esophageal pressure (pleural pressure) during the entire inflation phase of the tidal cycle. Calculations of end-expiratory *P*_TP_ when PEEP1 is applied will always tend to result in negative values. Our data revealed negative end-expiratory *P*_TP_ in all positions and conditions with PEEP1 (Figure 3); however, whether these negative values actually represent lung collapse is unclear. *P*_TP_ uses esophageal pressure as an estimate of global pleural pressure, and the esophageal balloon is influenced by anatomic factors that are not present elsewhere in the thorax. Thus, a negative calculated *P*_TP_ may relate to actual collapse of lung units, to simple reduction of air volume, or to regional early airway closure with subsequent air trapping. In the latter setting, true regional alveolar pressure actually exceeds that measured from the airway opening at end-exhalation. Such regional gas trapping has been shown to occur in obese normal humans who breathe in the horizontal supine position without PEEP [14]. Whatever the actual explanation might be, in horizontal positions, the driving pressures calculated from airway pressures alone (as traditionally done) have the potential to mislead [4].

Based upon our data for PEEP1 and non-PLEF conditions, we believe that while regional ‘lung collapse’ might be a plausible (if not unique) interpretation for negative end-expiratory *P*_TP_ in the supine position, tissue collapse is less likely to explain negative *P*_TP_ for the prone and semi-Fowler's positions in the same setting. The coexistence of negative *P*_TP_ and aerated lung at end-expiration also points toward the *local* characteristics of the pressures sensed by the esophageal catheter. The absolute *P*_ES_ may not represent overall pleural pressure, but it remains the most practical tool available to estimate pleural pressure in the clinical setting [15].

### Unilateral PLEF and lateral positioning

In designing this experiment, we questioned whether any benefit predictably accrues to adopting a specific lateral position when right or left PLEF is present. Surprisingly, our data showed little difference among overall FRC values in any horizontal position, regardless of the laterality of PLEF. Physically, there are four main vectors of compressive pressure influencing the lower lung when adopting lateral position: (1) abdominal content shift, (2) restriction of the dependent chest wall due to its contact with the bed, (3) gravitational shift of the mediastinal contents, and (4) weight of the contralateral hemithorax (lung ± pleural liquid). This rationale encouraged us to expect important differences between FRC and *P*_TP_ with changes of lateral position. However, our data showed inconsequential differences between right and left lateral positions, whatever the PLEF laterality. The unchanging global FRC strongly implicates the interdependence of forces across the various extrapulmonary compartments that envelop the lung. Apparently, in this experimental animal model, there is a balance between the upper ‘decompression’ and lower ‘compression’ of the lungs, which makes the laterality of the PLEF irrelevant when adopting different horizontal positions.

### Transpulmonary pressure and collapse

Either inflating the lung with positive pressure during tidal breathing or adding 10 cm H_2_O PEEP caused the calculated *P*_TP_ to convert from negative to neutral or positive values in all horizontal positions, with or without PLEF. Such conversion suggests the reopening of alveoli within the vicinity of the esophageal balloon and has been advocated as a marker by which to determine the PEEP needed to maintain recruitment and avoid regional tissue collapse in ARDS [3]. When supine, the vulnerability of *P*_ES_ to compression under mediastinal weight (relieved by lung expansion at PEEP10) might offer an alternative explanation of *P*_TP_ behavior in response to PEEP that is less tightly linked or even uncoupled from airspace closure [14]. While ‘cardiac weight artifact’ certainly has plausibility for that supine posture, this possibility seems less likely to apply in lateral and prone positions [16]. Assuming that *P*_ES_ reflects pleural pressure accurately, an alternative explanation could be that gas trapping occurs in horizontal positions at PEEP1 [12]. When gas trapping occurs, true alveolar pressure could be substantially higher than the 1 cm H_2_O airway pressure recorded through the central airway at end-exhalation (causing artifactually reduced *P*_TP_). Reversal of air trapping by inflation or by PEEP would raise measured *P*_TP_ into the observed positive range. Again, because calculated end-expiratory *P*_TP_ was negative in all positions (including prone and Fowler), this potential explanation, though attractive for the supine position, seems less compelling for the other positions tested.

### Transpulmonary pressure and FRC

Data are just not available regarding how well our only noninvasive estimator of *P*_TP_ tracks FRC when the lungs are asymmetrically affected. Our data demonstrate a discrepancy between *P*_TP_ calculations and global FRC changes in the setting of unilateral mechanical asymmetry. While negative end-expiratory *P*_TP_ was observed in all positions and conditions with PEEP1, FRC values in semi-Fowler's position were significantly higher when compared with those in horizontal positions. As indicated by these results, as well as by those reported in our previous experience with intra-abdominal hypertension (IAH) [4], the insensitivity of *P*_TP_ to changes on global FRC suggests the regional nature of *P*_ES_ measurements. Whether *P*_ES_ may be more accurate in reflecting regional changes on aeration distribution within the areas more vulnerable to collapse (e.g., the dependent lung units in proximity to the balloon) is unclear. Regional distribution changes of lung aeration and its correlation with *P*_TP_ merit further investigation.

### Limitations

As with all models, strong reservation is appropriate before translating these results to analogous clinical situations. While we believe that the principles elucidated here are qualitatively valid, the chest wall contours and lung compliance of pigs clearly differ from those of diseased humans, so that the magnitudes of the observed effects of position and PEEP may not tightly correspond. We studied a single tidal volume and two discreet levels of PEEP; quantitative effects would be influenced by the parameters chosen. Additionally, we cannot be sure that full recruitment was accomplished in every position, and it is clear that atelectasis of dependent lung units recurs quickly - even after a successful maneuver. The recruitment maneuvers performed in our study were based on our previous experience in comparing different recruitment maneuvers in three experimental models of acute lung injury [11]. Finally, interactive behaviors of the lung and chest wall are almost certain to be modified strongly by spontaneous breathing efforts. Although global measures of lung behavior (FRC and *P*_TP_) were less affected by horizontal positional change, this insensitivity does not imply that positional changes are inconsequential for gas exchange or breathing effort. Indeed, regional alterations visualized by CT suggest that customary global measures of bedside mechanics need to be complemented by techniques that are sensitive to the underlying components.

## Conclusions

In summary, the main points of interest resulting from this work are the following:

1. Despite induced nonsymmetry of regional mechanics and aeration caused by unilateral PLEF, FRC was not materially different among supine, prone, and lateral decubitus variations of the horizontal position. Rotation along the sagittal plane was required to raise global FRC.
2. Measured *P*_TP_ proved insensitive to mechanical asymmetry and to variations of regional gas volume caused by varying position.
3. With PEEP1 applied, calculated end-expiratory *P*_TP_ was negative in all positions; applying PEEP10 caused a transition to positive end-expiratory *P*_TP_.

This study was conducted with approval from and under the supervision of our institution's animal care and use committee and has therefore been performed in accordance with the ethical standards laid down in the 1964 Declaration of Helsinki and its later amendments.

## Authors’ original submitted files for images

Below are the links to the authors’ original submitted files for images. Authors’ original file for figure 1 Authors’ original file for figure 2 Authors’ original file for figure 3 Authors’ original file for figure 4
