# Supplementary figures and images for: Unilateral mechanical asymmetry: positional effects on lung volumes and transpulmonary pressure
Source: Intensive Care Med Exp. 2014 Feb 5;2:4. doi: 10.1186/2197-425X-2-4 (PMC4513031; doi:10.1186/2197-425X-2-4)

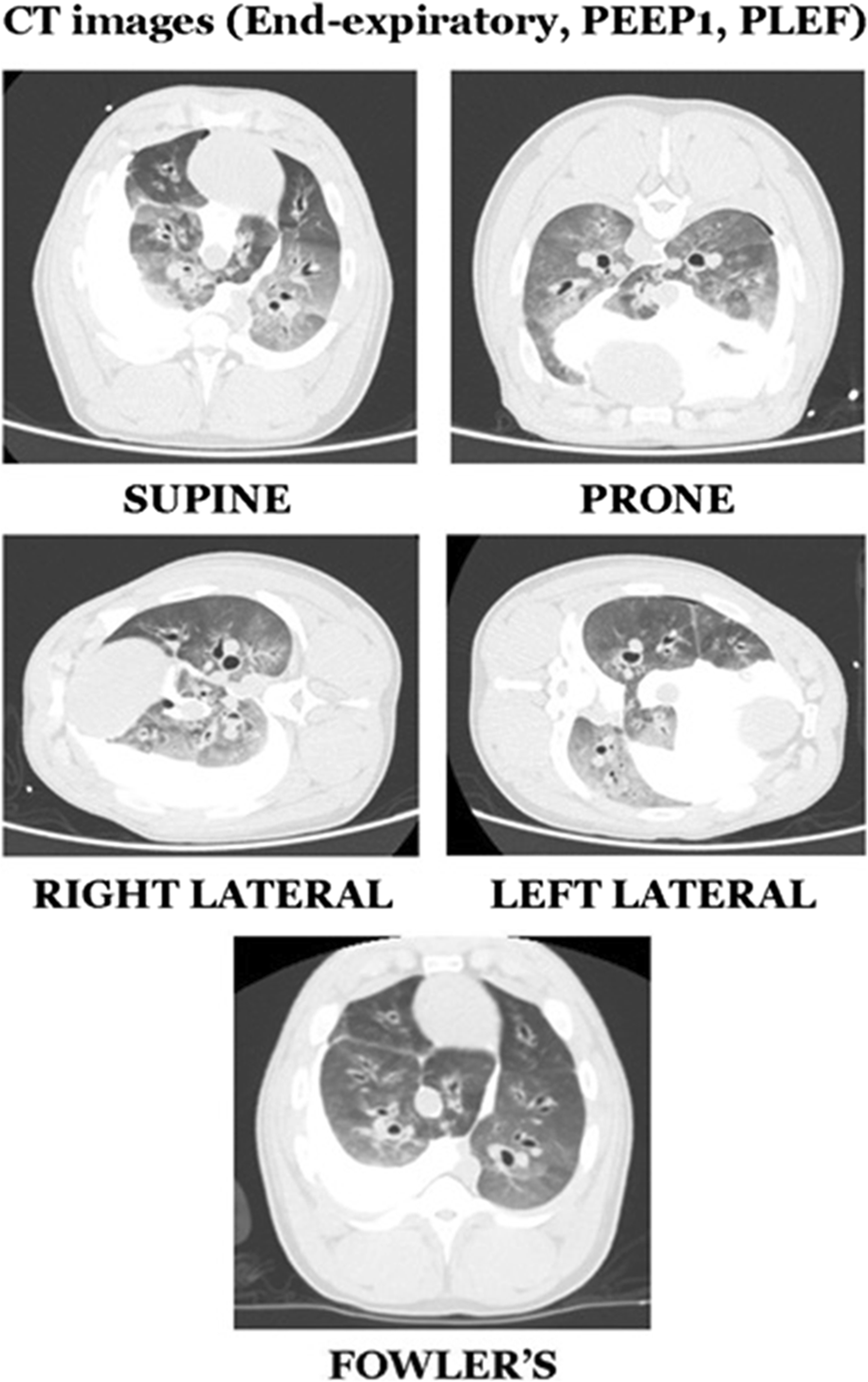

Supplement: Supplementary file 1 — Authors’ original file for figure 1 [file 40635_2013_3_MOESM1_ESM.tiff]

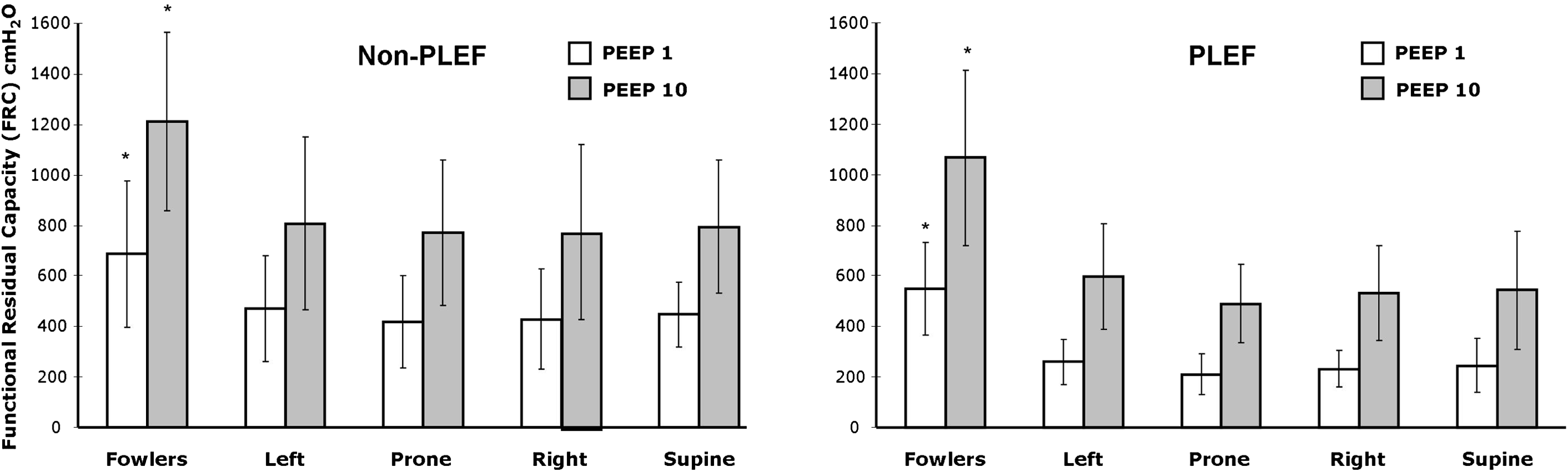

Supplement: Supplementary file 2 — Authors’ original file for figure 2 [file 40635_2013_3_MOESM2_ESM.tiff]

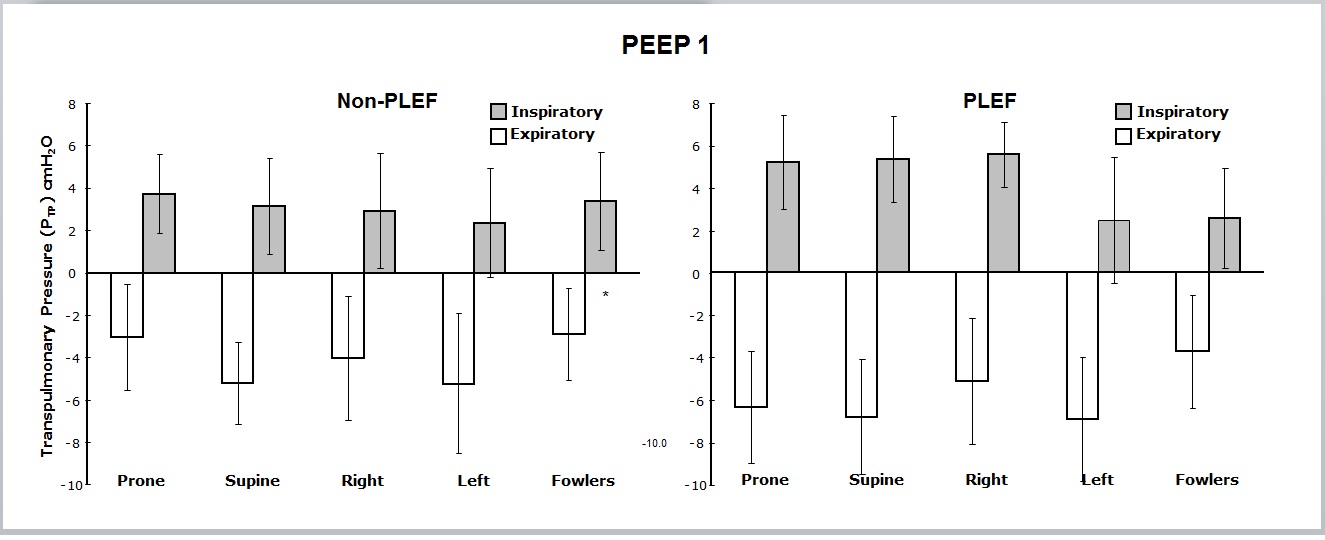

Supplement: Supplementary file 3 — Authors’ original file for figure 3 [file 40635_2013_3_MOESM3_ESM.jpeg]

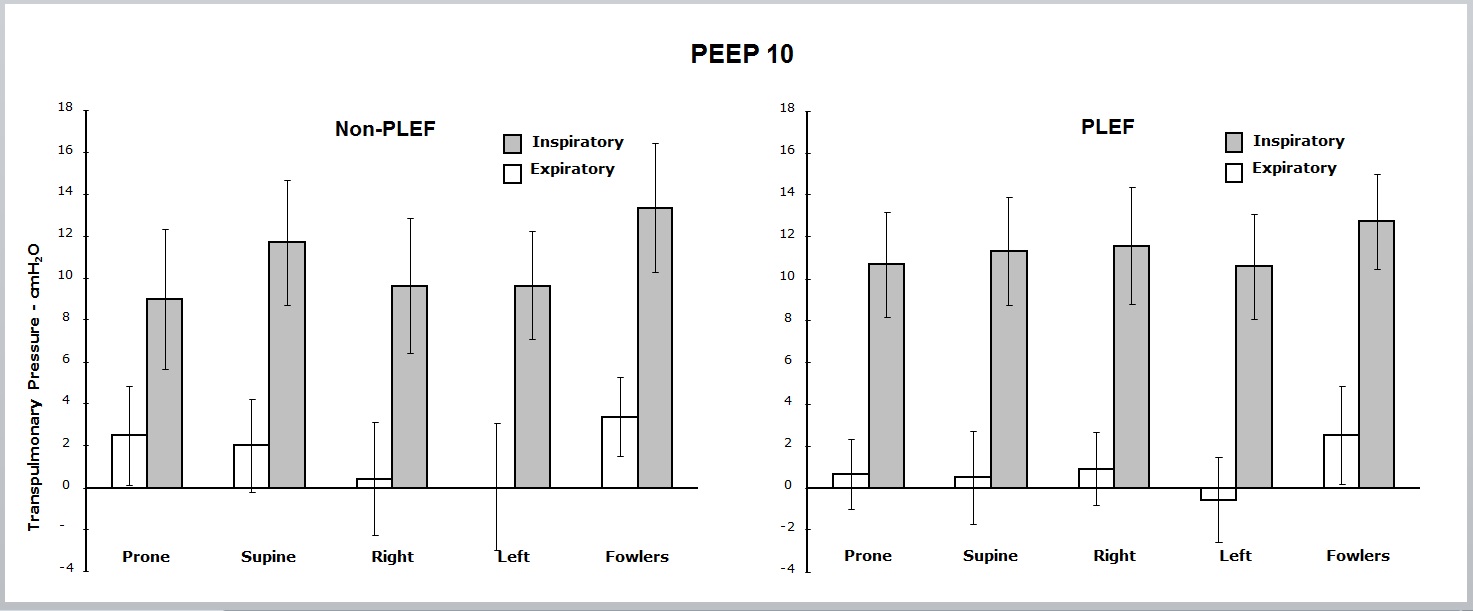

Supplement: Supplementary file 4 — Authors’ original file for figure 4 [file 40635_2013_3_MOESM4_ESM.jpeg]
